# Nationwide Remapping of *Schistosoma mansoni* Infection in Rwanda Using Circulating Cathodic Antigen Rapid Test: Taking Steps toward Elimination

**DOI:** 10.4269/ajtmh.19-0866

**Published:** 2020-05-18

**Authors:** Eugene Ruberanziza, Udo Wittmann, Aimable Mbituyumuremyi, Alphonse Mutabazi, Carl H. Campbell, Daniel G. Colley, Fiona M. Fleming, Giuseppina Ortu, Govert J. van Dam, Irenee Umulisa, Jamie Tallant, Michee Kabera, Muhammed Semakula, Paul L. A. M. Corstjens, Tharcisse Munyaneza, Warren Lancaster, Jean Bosco Mbonigaba, Michelle N. Clements

**Affiliations:** 1 Neglected Tropical Diseases and Other Parasitic Diseases Unit, Rwanda Biomedical Center, Ministry of Health, Kigali, Rwanda;; 2 Malaria and Other Parasitic Diseases Division, Rwanda Biomedical Center, Ministry of Health, Kigali, Rwanda;; 3 SCI Foundation, London, United Kingdom;; 4 Consult A.G. Statistical Servicesc, Zurich, Switzerland;; 5 Vector Control Unit, Rwanda Biomedical Center, Ministry of Health, Kigali, Rwanda;; 6 Schistosomiasis Consortium for Operational Research and Evaluation, Center for Tropical and Global Emerging Diseases, University of Georgia, Athens, Georgia;; 7 Department of Microbiology, University of Georgia, Athens, Georgia;; 8 Leiden University Medical Center, Leiden, Netherlands;; 9 African Leaders Malaria Alliance (ALMA), National Institute of Medical Research Complex, Dar-es-Salam, Tanzania;; 10 The END Fund, New York, New York;; 11 Epidemiology Unit, Rwanda Biomedical Center, Ministry of Health, Kigali, Rwanda;; 12 HIV/AIDS and STIs Division, Rwanda Biomedical Center, Ministry of Health, Kigali, Rwanda;; 13 Microbiology Unit, National Reference Laboratory (NRL) Division, Rwanda Biomedical Center, Ministry of Health, Kigali, Rwanda;; 14 MRC Clinical Trials Unit, University College London, London, United Kingdom

## Abstract

The field standard for the detection of *Schistosoma mansoni* infection is Kato–Katz (KK), although it misses many active infections, especially light infections. In 2014, a reassessment of *S. mansoni* prevalence was conducted in Rwanda using the more sensitive point-of-care circulating cathodic antigen (POC-CCA) rapid assay. A total of 19,371 children from 399 schools were selected for testing for single urine CCA. Of these, 8,697 children from 175 schools were also tested with single stool double-slide KK. Samples from eight of these 175 schools were tested again with CCA and additionally with the highly specific and sensitive up-converting phosphor-lateral flow circulating anodic antigen (UCP-LF CAA) assay. Latent class analysis was applied to all four test results to assess sensitivity and specificity of POC-CCA and estimate the proportion of trace results from Rwanda likely to be true infections. The overall prevalence of *S. mansoni* infection in Rwanda when CCA trace results were considered negative was 7.4% (school interquartile range [IQR] 0–8%) and 36.1% (school IQR 20–47%) when trace was considered positive. Prevalence by KK was 2.0% with a mean intensity of infection of 1.66 eggs per gram. The proportion of active infections among children diagnosed with CCA trace was estimated by statistical analysis at 61% (Bayesian credibility interval: 50–72%). These results indicate that *S. mansoni* infection is still widespread in Rwanda and prevalence is much underestimated by KK testing. Circulating cathodic antigen is an affordable alternative to KK and more suitable for measuring *S. mansoni* prevalence in low-intensity regions.

## INTRODUCTION

Worldwide, an estimated 290 million of the world’s most impoverished people are affected by schistosomiasis,^1^ whereas over 779 million people live in areas of high risk for the transmission of schistosomes.^2,3^ The WHO guidelines for control of morbidity due to helminth infections and their elimination^4^ are being implemented by national programs across Africa in collaboration with bilateral and nongovernmental agencies and donations of drugs by pharmaceutical companies. The mapping and detection of *Schistosoma mansoni* infections for national programs has been primarily accomplished using the Kato–Katz (KK) method of microscopic stool examination. This field and laboratory assay detects parasite eggs to estimate prevalence and intensity of infection and ultimately determines the treatment strategy of a program.^5,6^ Kato–Katz requires the collection of stool and, although considered highly specific, is known to be relatively insensitive, especially in areas with low prevalence and intensity.^7–9^ Recently, the development and evaluation of a urine-based, field-friendly, POC assay that detects worm-produced CCA has shown to be relatively specific and highly sensitive across endemic settings,^7–9^ although there have been difficulties in interpreting the so-called trace results lying between clearly negative and clearly positive. Point-of-care circulating cathodic antigen (POC-CCA) is particularly valuable for national programs to evaluate the burden of *S. mansoni* where regular preventive chemotherapy with praziquantel has been ongoing and there is, consequently, lower endemicity and where program goals may change to elimination as a public health problem.^10–12^ The up-converting phosphor-lateral flow circulating anodic antigen assay (UCP-LF CAA or CAA), which detects a different adult worm antigen from CCA, is a laboratory-based assay that has been shown to be both highly sensitive and specific for the detection of worms because of the excretion in the urine of the circulating anodic antigen and the use of the UCP reporter system.^13,14^

In 2007, the Rwanda Ministry of Health, in collaboration with its partners, established the neglected tropical disease control program. Following the initial baseline mapping conducted in 2007–2008 using the KK technique, the national program commenced regular mass drug administration (MDA) against schistosomiasis and soil-transmitted helminthiasis (STH) as per the WHO guidelines.^15,16^ According to the baseline mapping and additional studies in Rwanda, nine of 30 districts of the country were identified as at risk of intestinal schistosomiasis.[Bibr b16][Bibr b18] Based on these survey data, the program implemented annual MDA of praziquantel for school-age children in all endemic areas with at least 10% prevalence. In addition, adults were treated between 2007 and 2013 in areas where the prevalence was found to be at least 30%.^18^

From June 2014 to mid-July 2014, a national school-based reassessment was carried out, using both KK for the detection of *S. mansoni* eggs and POC-CCA for detection of the worm antigen to determine the feasibility of moving toward schistosomiasis elimination in Rwanda. In addition, after the surveys were completed, urine samples from children in a purposively selected subset of eight schools assessed by both POC-CCA and KK were tested in a laboratory setting, repeating the POC-CCA assay and also further evaluating by CAA.

In this article, we

1. present the results from the 2014 POC-CCA mapping of 388 schools;
2. compare the POC-CCA and KK results in 175 schools evaluated by both tests; and
3. compare the sensitivity and specificity of POC-CCA, KK, and CAA in eight schools purposively selected for additional assays.

## MATERIALS AND METHODS

### Study site.

Rwanda is a landlocked country with 23 lakes, the main being Lake Kivu, Lake Muhazi, Lake Ihema, Lake Bulera, Lake Ruhondo, and Lake Mugesera, and numerous rivers.^19^ Rwanda is divided into 30 districts, with each district being divided into a number of subdistricts, termed sectors. The 2012 census estimated the population to be 10.5 million people, 48% of which are below the age of 18 years. Approximately 70% of the children aged between 3 and 17 years are attending school.^20,21^

### Study design.

#### Mapping units.

**Figure 1.**
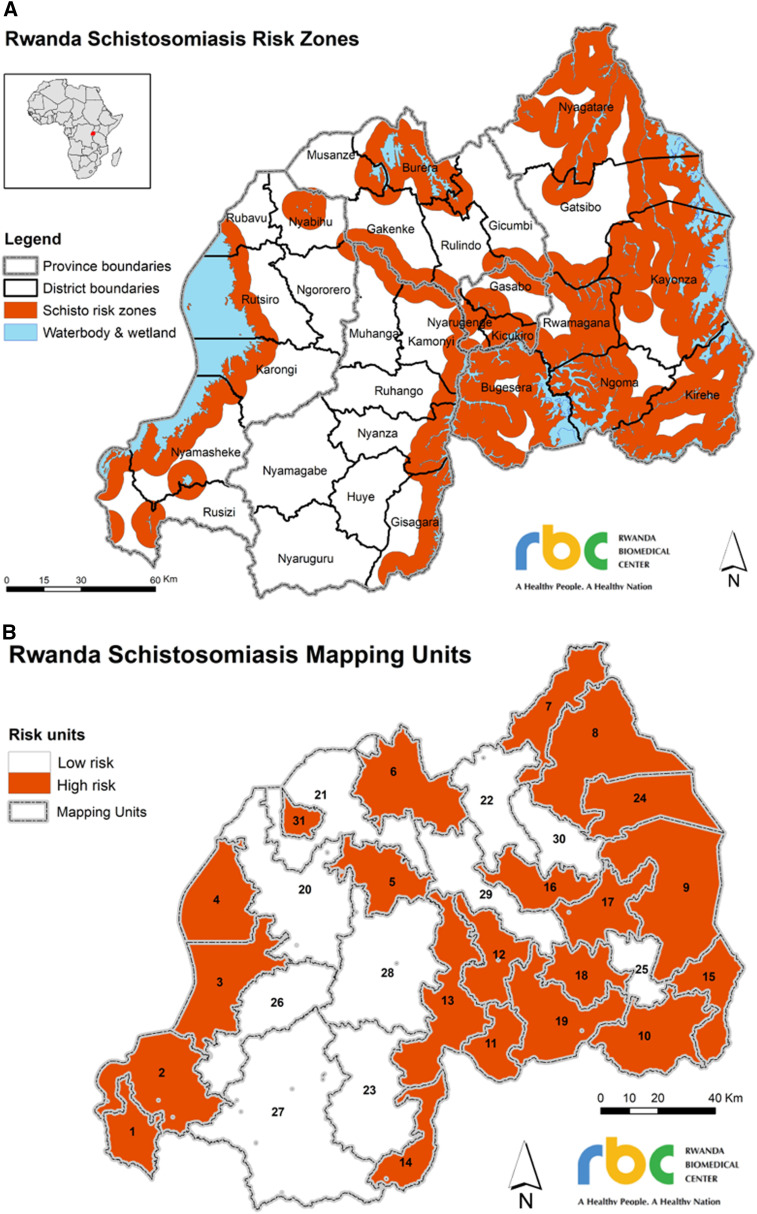
Creation of Rwanda mapping units for *Schistosoma mansoni* infections. Five-kilometer buffer zones were created around permanent water bodies and wetlands (as far as information was available), shown in orange in the top of the figure (**A**). Contiguous groups of sectors inside the same 5-km buffer zone were grouped into high-risk mapping units, whereas contiguous sectors with little or no area in buffer zones were grouped into low-risk mapping units. The bottom figure (**B**) shows the distribution of high- and low-risk mapping units.

A multistage, cross-sectional, cluster-randomized survey was undertaken to estimate prevalence within independent mapping units. Each “mapping unit” consisted of a number of sectors that were likely to share transmission characteristics based on previous schistosomiasis prevalence data and eco-epidemiological settings.^22^ A 5-km buffer zone surrounding perennial water bodies and wetlands was drawn (Figure 1A), and contiguous groups of sectors inside the same 5-km buffer zone were grouped into high-risk mapping units, whereas contiguous sectors with little or no area in buffer zones were grouped into low-risk mapping units. Location information of wetlands was only used as available. Based on this system, 10 mapping units at low risk and 21 mapping units at high risk of *S. mansoni* were identified (Figure 1B).

### Sample size and sampling procedures.

Schools were randomly selected within each mapping unit. Sample size calculations assumed prevalence of ∼10% by KK and 50% by POC-CCA^9^ in high-risk units, and 5% by KK and 20% by POC-CCA in low-risk units and a school-level intra-class correlation coefficient of 0.13 based on estimates from the 2008 mapping data. To estimate the prevalence with an absolute margin of error of 10% on a 95% CI, 13 schools in each high-risk mapping unit and nine schools in each low-risk mapping unit were randomly selected. We did not adjust for population size (e.g., finite population correction) as less than 5% of the population of Rwanda was surveyed.^23^

In addition to the 363 randomly selected schools, 31 proposed sentinel schools were also included: 12 former and 19 new sentinel schools. Sentinel schools have been used by the control program to monitor infection levels over time. The former sentinel schools had been monitored since 2008 and were in five districts of Burera, Musanze, and Gicumbi in Northern Province, Nyagatare in Eastern Province, and Rutsiro in Western Province, to allow the program to be evaluated in two low-, two medium-, and two high-prevalence schools. The new sentinel schools were selected from areas historically considered to be “hotspots” based on epidemiological surveys previously carried out in the primary schools.^18,22,24^ The selection of schools for testing with KK was based on mapping for STHs requiring five to seven schools per district, and schools were selected for mapping with both tests where there was overlap in requirements.

A total of 400 schools were selected for mapping—175 schools were selected for testing with both POC-CCA and KK, 213 were selected for testing with POC-CCA only, and 12 were selected for testing with KK only. However, data were not collected at one school (selected for KK testing only) as there were very few pupils in the target age range of 13–14 years.

#### Selection of samples for assessment with POC-CCA and UCP-LF CAA.

The urine samples of children from eight purposively selected schools which had been assessed by POC-CCA and KK were sent to the Leiden University Medical Center where they were assessed again by POC-CCA and additionally by the UCP-LF CAA assay. The sample size of eight schools was mainly driven by cost considerations, and schools were selected following review of the KK and POC-CCA results. Schools selected were those that had sufficient numbers of urine samples from children who were 1) positive by KK and from children who were 2) negative by KK but positive by POC-CCA. Assessment of trace POC-CCA results was a priority; thus, efforts were made to include an adequate number of children who had trace-positive POC-CCA readings.

### Data collection.

#### Kato–Katz and POC-CCA data within schools.

Within each school, 50 pupils aged 13–14 years with equal numbers of boys and girls were randomly selected from all pupils in the target age range present in school on the day of sampling. If less than 50 pupils were present in school, 10- to 16-year-olds were also included in the sample.

Point-of-care circulating cathodic antigen testing was performed on a single urine sample from each child according to the producer’s instructions (Rapid Medical Diagnostics, Pretoria, South Africa; batch 33955; expiry date: June 2015). Results were graded: incremental band intensities as negative, trace, and three gradients of positive results.^25^ In addition, each urine sample was immediately examined for macroscopic hematuria as a potential marker for urinary schistosomiasis infection.^2^ Kato–Katz testing used a single stool specimen from each child, processed to provide two slides for microscopy.^4,5^

Quality assurance of POC-CCA was ensured through comparison with an image of the five POC-CCA test results, and each result was verified by an independent reviewer (either a technician from the national reference laboratory or an external consultant). For KK, each slide was read by two different technicians, and discordant results were adjudicated by an independent reviewer, who also verified 10% of the slides each day.

Data collection was carried out on paper forms where POC-CCA and KK data were collected in schools and via smartphones in schools where only POC-CCA was used. Data collected on paper forms were manually double-entered into an Excel database by data clerks. Data inputted through smartphones were outputted to Excel.

All children who tested positive for intestinal schistosomiasis by either the POC-CCA or KK assay were treated with praziquantel (PZQ; 40 mg/kg administered by dose pole) and those positive for any STH were treated with 400 mg of albendazole.

#### Point-of-care circulating cathodic antigen and UCP-LF CAA assays processed in Leiden.

The urine samples from the eight selected schools were anonymized, blinded, and sent to Leiden by a commercial shipper, stored at a constant temperature of 4°C throughout transit. In Leiden, the samples were stored at −20°C before being independently tested by POC-CCA and UCP-LF CAA. The technicians running the tests were blinded to the results obtained in Rwanda. The POC-CCA test (batch 50174; expiry date: May 2017) was performed as described earlier and scored visually in a semiquantitative way using the scoring system as aforementioned. The UCP-LF CAA assay for urine was performed with 2 mL urine (the UCAA2000 assay format).^12,26^ Briefly, 2 mL urine was extracted with an equal volume of 4% (w/v) trichloroacetic acid. A centrifugal filtration device (Amicon^®^ Ultra-4, Millipore) was used to concentrate the resulting supernatants. The resulting clear supernatant was concentrated using Amicon centrifugal filtration devices (Amicon Ultra-4, Millipore) to a final volume of 20–30 μL, of which 20 μL was analyzed on UCP-LF CAA test strips using the wet reagent format. A standard series of known CAA concentrations spiked in negative urine established CAA concentrations in the test samples. As a cutoff threshold, 0.1 pg CAA per ml urine was used, whereas 150 pg/mL was considered as the highest CAA concentration that could be quantitatively measured because of the very high UCP signals.

### Statistical analysis.

#### Circulating cathodic antigen mapping results and comparison with KK.

When POC-CCA trace readings were taken as test-positive results, the results were recorded as “POC-CCA trace positive”; when taken as test negative, the results were recorded as “POC-CCA trace negative.” For all tests, prevalence was defined as the number of children with a positive result, divided by the number of all tested children. Statistical analysis was performed under R version 3.4.0.^27^

*Schistosoma mansoni* eggs per gram (epg) of stool from KK were calculated for each slide by multiplying the number of eggs per slide by 24. The mean intensity of infection for each individual was the arithmetic mean of the epg over both slides where two slides were recorded or just the one observation if only one slide was recorded. Light-intensity *S. mansoni* infection was defined as 1–99 epg, moderate-intensity infection as 100–399 epg, and heavy-intensity infection as > 399 epg.^4^

Prevalence of *S. mansoni* infection based on POC-CCA was analyzed with logistic mixed models. Standardized age (subtracting the mean and dividing by the SD), its associated quadratic, gender, and mapping units were used as fixed effects, with school as the only random effect. We used the R-package lme4^28^ with different optimizers and the package glmmADMB^29^ to obtain robust results. To investigate the effect of proximity to perennial water bodies, the risk status of the mapping unit was added as fixed factors and mapping unit as a random factor to the aforementioned model.

#### Estimating sensitivity and specificity of tests.

To assess sensitivity and specificity of the POC-CCA test, we analyzed CCA trace considered as negative and positive separately. Each latent class analysis (LCA) included results from four tests—POC-CCA collected in Rwanda (CCAR), KK CCAR, POC-CCA collected in Leiden (CCAL), and UCP-LF CAA CCAL. Full details of the LCA are available elsewhere.^12^

A Bayesian framework under OpenBUGS 3.2.3 ^31^ was used to estimate the true prevalence of *S. mansoni* infections at each of the eight schools together with the sensitivity and specificity of each of the four diagnostic tests. For prevalence in each of the eight schools, independent beta priors on the uniform distribution (α = β = 1) were used. Priors for the sensitivity and the specificity of each of the test used the epi.betabuster function from the R-package epiR.^32^ The prior for the specificity of KK was the strongest with a mode at 95% and 95% of its values greater than 80%, reflecting the high known specificity of KK, and Supplemental Table S1 lists the parameters of the beta distribution for each of the test priors. We ran the models using three chains, each with 2000 iterations, a thin of 40, and a burn-in of 200, resulting in an output of 5,400 iterations.

For each pair of tests, the covariances between their sensitivities and their specificities were added to the likelihood equations. We started by adding the covariance terms for all six possible pairings of tests but ran into convergence problems. Consequently, we fitted models with covariance terms for each pair of tests separately (adding in sensitivity and specificity terms) and compared the deviance information criterion (DIC) of the resulting models (Supplemental Table S2). We selected the two models with the lowest DIC and tested any additional covariance terms that resulted in a lower DIC. We also attempted to fit the trace-positive and the trace-negative results in one model, but this model did not converge. We used uniform priors for the covariance components with limits chosen for probabilities to remain bounded between zero and one. Model fit was assessed by comparing estimated and observed prevalence from each test, with large differences indicating the model has converged to a local rather global maximum. Gelman diagnostics^33^ were also used to assess the convergence of the models.

Overall infection prevalence was the average of the estimated school prevalence weighted for the number of children sampled in each school. A simulated distribution for each test prevalence was obtained from the posterior distributions of the true prevalence and the sensitivity and specificity of each test. This was used to compare the overall infection prevalence to each of the test prevalences.

An estimate of the proportion of CCA trace results that were truly positive was obtained by combining the positive predictive values (PPVs) of CCA from each model. Full details are available in Clements et al.^12^

### Ethical considerations.

This survey was approved by the Institutional Review Board of Imperial College London (St Mary Research Ethics Committee of Imperial College, United Kingdom, 2003/EC No 03.36, R&D No: 03/SB/003E, amended in 2007/REC Ref: AM01, May 2007) and by the Rwanda National Ethics Committee (RNEC) (approval letter no. 261/RNEC/2014).

School teachers were contacted to communicate to parents and guardians about the survey and to get permission for including their children for testing and treating if found positive. During data collection, written consent was signed by school directors, parents, and guardians, as well as children (assent), before their participation to the screening. It was explained to the children that they were free to refuse provision of urine and stool samples if they did not want to provide them. All data were stored in purpose-built MS Excel files with no names but only ID numbers.

## RESULTS

### Point-of-care circulating cathodic antigen mapping results.

**Figure 2.**
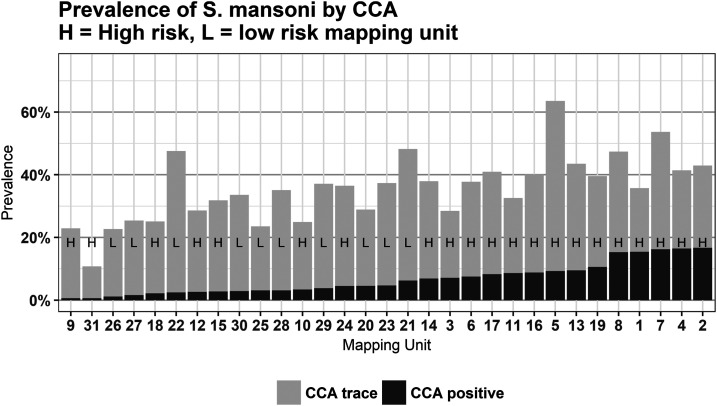
Prevalence of *Schistosoma mansoni* by point-of-care circulating cathodic antigen (POC-CCA) results and by mapping unit. H and L denotes mapping unit as high- or low-risk units of *S. mansoni*, respectively, as determined before the survey. Prevalence by POC-CCA trace negative can be read off the top of the black bars, whereas prevalence by POC-CCA trace positive can be read off the top of the grey bars. Mapping units were sorted by prevalence, low to high, with trace as negative.

Overall prevalence using POC-CCA was 7.4% when trace results were considered negative (POC-CCA trace negative) and 36.1% when trace results were considered positive (POC-CCA trace positive; Table 1). At the mapping unit level, POC-CCA trace-negative prevalence ranged from 0.6% to 16.7%, with a median of 4.7%, and POC-CCA trace-positive prevalence ranged from 10.8% to 63.5%, with a median of 36.5% (Figure 2; see Supplemental Table S3 for prevalence estimates in each mapping unit). Of all schools surveyed, at least one child had a positive result in 70.6% of schools by POC-CCA trace negative, rising to 99.2% by POC-CCA trace positive.

**Table 1:** Summary statistics from the three different datasets analyzed

Dataset		POC-CCA mapping	POC-CCA and KK comparison	Assessment with CAA in Leiden
Number of schools		388	175	8
Number of pupils		19,371	8,697	396
Age of pupils (years), mean (SD)		13.4 (0.75)	13.3 (0.75)	13.4 (0.67)
Percentage female		50.1	49.9	50.0
Prevalence, % (school IQR)	CCA trace negative in Rwanda	7.4 (0.0–8.0)	8.6 (1.0–8.0)	31.3 (32.0 –37.1)
	CCA trace positive in Rwanda	36.1 (20.0–47.0)	37.5 (22.0–49.0)	65.7 (64.0–78.5)
	Kato–Katz in Rwanda	–	2.0 (0.0–0.0)	8.1 (3.0–11.2)
	CCA trace negative in Leiden	–	–	33.8 (25.5–47.8)
	CCA trace positive in Leiden	–	–	55.8 (50.0–75.0)
	CAA in Leiden	–	–	44.2 (31.0–63.5)

IQR = interquartile range; KK= Kato–Katz; POC-CCA = point-of-care circulating cathodic antigen. The 175 schools assessed by POC-CCA (CCA) and KK were a subset of the 388 schools assessed using POC-CCA, and the eight purposively selected schools also assessed by POC-CCA and CAA in Leiden were a subset of the 175 schools assessed with POC-CCA and KK in Rwanda. Variability between schools was reported as IQR of the school prevalence estimates. Note that 81% of schools sampled recorded no infections by KK.

Prevalence by POC-CCA was lowest in mapping unit 31, at 0.62% by POC-CCA trace negative and 10.77% by POC-CCA trace positive (Supplemental Table S3). We compared the other mapping units with mapping unit 31 to determine regions with significantly higher prevalence of *S. mansoni* infections. By POC-CCA trace negative, five mapping units (9, 18, 22, 26, and 27) were not significantly different from the prevalence of the reference unit, whereas by POC-CCA trace positive, all mapping units had significantly higher prevalence to the reference mapping unit. The risk of being infected with *S. mansoni* was significantly lower for pupils from low-risk mapping units only by POC-CCA trace negative (OR 0.46, *P* = 0.03; Table 2), but there was no significant difference in prevalence between high and low mapping units by POC-CCA trace positive (OR 0.90, *P* = 0.62; Table 2). For both models, but especially for the POC-CCA trace positive model, the *R*^2^ marginal value was low (0.02 and 0.001; Table 2), indicating that the fixed effects did not explain the variation in the data well.

**Table 2:** Modeling the prevalence of *Schistosoma mansoni* infections controlling for gender, age, and the risk status of the mapping unit, that is, proximity to perennial water body

		POC-CCA trace negative			POC-CCA trace positive		
		19,317 pupils, 388 schools			19,317 pupils, 388 schools		
All POC-CCA mapping schools		*R*^2^ marginal = 0.022, *R*^2^ conditional = 0.434			*R*^2^ marginal = 0.001, *R*^2^ conditional = 0.262		
Fixed effects	Levels	Parameter	Adjusted odds ratio	*P*-value	Parameter	Adjusted odds ratio	*P*-value
Intercept		−3.44 (0.18)	0.03 (0.02, 0.05)	< 0.001	−0.69 (0.12)	0.5 (0.4, 0.63)	< 0.001
Gender	F	–	–	–	–	–	–
	M	0.31 (0.06)	1.36 (1.21, 1.53)	< 0.001	0.08 (0.033)	1.09 (1.02, 1.16)	0.013
Age.s		0.04 (0.035)	1.04 (0.97, 1.12)	0.246	−0.03 (0.02)	0.97 (0.94, 1.01)	0.14
Age.s2		−0.05 (0.022)	0.95 (0.91, 0.99)	0.024	−0.01 (0.01)	0.99 (0.97, 1.01)	0.439
Risk	High risk	–	–	–	–	–	–
	Low risk	−0.7 (0.32)	0.5 (0.26, 0.94)	0.031	−0.11 (0.22)	0.90 (0.59, 1.38)	0.6247
Random effects							
		Variance	SD		Variance	SD	
Mapping unit (intercept)		0.43	0.66		0.21	0.46	–
School (intercept)		2	1.4		0.95	0.98	–

POC-CCA = point-of-care circulating cathodic antigen.

### Comparison of POC-CCA and Kato-Katz results.

Prevalence by POC-CCA in schools sampled by both POC-CCA and KK was 8.6% (mapping unit range [ur] = 0.5–32.3%; Table 1) by POC-CCA trace negative and 37.5% (ur = 7.0–66.4%) by POC-CCA trace positive. The prevalence measured by KK in these same schools was just 2% (ur = 0.0–9.4%), with the overall mean intensity of *S. mansoni* infections at 1.66 epg (ur = 0–10.11epg). Of all children diagnosed as positive by KK (*n* = 172), 75% had light infections, 22.7% had moderate infections, and only four children (2.3%) had heavy infections.

**Figure 3.**
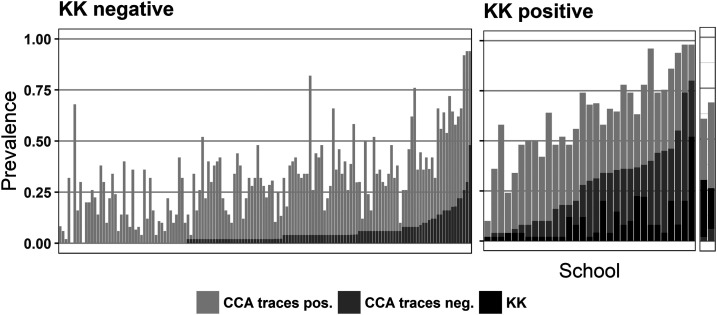
Prevalence of *Schistosoma mansoni* at each of the 175 schools assessed by Kato–Katz (KK) and point-of-care circulating cathodic antigen (POC-CCA). The left figure shows the prevalence measured by POC-CCA at all schools where prevalence by KK was 0% and the middle and right figures show the prevalence measured by KK and by POC-CCA from KK-positive schools, respectively. The schools are in the order of prevalence from POC-CCA with trace as negative. The two right-most bars represent schools where the prevalence by KK was greater than the prevalence by POC-CCA with trace taken as negative.

Of all 8,525 pupils who were negative by KK (i.e., no eggs were detected in stool samples), 63% were also negative by POC-CCA, 29.1% had trace-positive results, and 7.2% POC-CCA had results above trace. Conversely, of the 172 pupils positive by KK, 8% were POC-CCA negative, 18% were POC-CCA trace positive, and 74% had POC-CCA results above trace Figure 3.

### Estimating sensitivity and specificity of tests.

The eight purposively selected schools had, by design, higher prevalence by POC-CCA than the population of 175 schools: at 31.3% (school interquartile range [IQR] 32.0–37.1%) by POC-CCA trace negative and 65.7% (school IQR 64.0–78.5%) by POC-CCA trace positive (Supplemental Table S9). Similarly, the selected schools had higher prevalence by KK, with a prevalence of 8.1% (school IQR 3.0–11.2%) and a mean infection intensity of 5.58 epg (SD: 27.27).

Two POC-CCA tests were performed for the eight schools: one in Rwanda and one in Leiden. Of the 396 samples, 59% had the same POC-CCA result in both Rwanda and Leiden, 38% differed by one level, and just 3% differed by more than one level (Supplemental Table S10). Of the 163 samples with different POC-CCA results, 66% had a “trace” result in one test, with 42% of the trace results in Rwanda testing negative in Leiden and 22% of the trace results in Leiden testing negative in Rwanda. Both tests showed a similar prevalence by POC-CCA trace negative (Rwanda: 31.3%, Leiden: 33.8%; Supplemental Table S9). However, the percentage of trace-positive readings was higher in Rwanda at 34.3%, compared with 22.0% in Leiden, resulting in prevalence by POC-CCA trace positive of 65.7% in Rwanda and 55.8% in Leiden Table 3.

**Table 3:** Estimates of sensitivity and specificity of the four diagnostic tests applied to the urine samples from eight selected schools which were also assayed at the Leiden University Medical Center

	POC-CCA trace negative	POC-CCA trace positive
Sensitivity (%) (95% BCI)		
Kato–Katz in Rwanda	14.1 (8.3, 19.8)	2.5 (0.2%, 6.6%)
CCA in Rwanda	46.1 (35.6, 56.1)	82.5 (77.6%, 86.9%)
CCA in Leiden	94.2 (85.3%, 99.5%)	93.6 (89.0%, 97.3%)
CAA in Leiden	97.0 (90.6%, 99.8%)	90.5 (83.6%, 96.9%)
Covariance KK and CAA	0.6 (0.0%, 2.2%)	0.3 (0.0%, 1.0%)
Covariance CCAR and CCAL	7.0 (3.2%, 11.2%)	–
Specificity (%) (95% BCI)		
Kato–Katz in Rwanda	98.2 (95.0, 99.8)	98.9 (97.1%, 99.9%)
CCA in Rwanda	85.3 (80.4%, 90.6)	53.5 (50.0%, 57.6%)
POC-CCA in Leiden	85.9 (80.3%, 92.3%)	98.2 (94.4%, 99.9%)
CAA in Leiden	83.1 (77.9%, 87.7%)	49.2 (45.8%, 54.2%)
Covariance KK and CAA	12.1 (8.9%, 15.3%)	21.9 (19.2%, 23.8%)
Covariance CCAR and CCAL	10.3 (6.8%, 13.6%)	–
PPV of CCA in Rwanda (%) (95% BCI)		
Trace as negative	68.3 (58.0, 78.5)	–
Trace as positive	64.5 (59.9%, 68.9%)	–
Trace	61.6 (50.1%, 72.1%)	–

BCI = Bayesian credibility interval; POC-CCA = point-of-care circulating cathodic antigen.

The test with the lowest sensitivity was KK, at 14.1% (Bayesian credibility interval [BCI]:8.3–19.8%) by POC-CCA trace negative and just 2.5% (BCI: 0.2–6.6%) by POC-CCA trace positive (Table 4). The highest estimates of sensitivity were for CAA in Leiden when trace was considered negative (97.0%; BCI: 90.6–99.8%) followed by POC-CCA in Leiden when trace was considered negative (94.2%; BCI: 85.3–99.5%). Specificity estimates were highest for KK, at 98.9% (BCI: 97.1–99.9%) and for CCA in Leiden again at 98.2% (BCI: 94.4–99.9%) both from the trace-positive model.

**Table 4:** Infection prevalence estimated by the latent class analysis and the differences to the prevalences estimated by each diagnostic test

	Trace negative				Trace positive			
	Mean	SD	LBCI	UBCI	Mean	SD	LBCI	UBCI
Infection prevalence (%)	40.7	2.5	35.8	45.8	50.6	2.0	46.7	54.5
Estimated prevalence (%)								
Kato–Katz in Rwanda	8.1	1.3	5.6	10.9	8.1	1.3	5.6	10.9
POC-CCA in Rwanda	31.3	2.2	27.0	35.6	65.7	1.9	61.9	69.4
POC-CCA in Leiden	33.8	2.1	29.8	38.1	55.8	2.1	51.8	59.8
CAA in Leiden	44.3	2.1	40.2	48.5	44.2	2.1	40.2	48.2
Estimated test—disease prevalence (%)								
Kato–Katz in Rwanda	−32.6	2.9	−38.3	−27.1	−42.5	2.4	−47.2	−37.8
POC-CCA in Rwanda	−9.4	3.4	−16.1	−2.7	15.1	2.8	9.6	20.6
POC-CCA in Leiden	−6.9	3.3	−13.4	−0.5	5.3	2.9	−0.2	11.0
CAA in Leiden	3.6	3.3	−2.9	10.2	−6.4	2.9	−11.7	−0.7

Comparison of model outputted prevalence with observed prevalence showed that the model estimated the prevalence by POC-CCA in Rwanda and KK satisfactorily. However, prevalence estimates by POC-CCA in Leiden were 46.7% for trace negative and 48.2% for trace positive, whereas the corresponding observed prevalence were 33.8% and 55.8%, respectively. In addition, the model estimate of the CAA prevalence from the trace-positive model was 70.9%, whereas the observed CAA prevalence was only 44.2%. Using different priors and covariance terms did not fix these issues.

**Figure 4.**
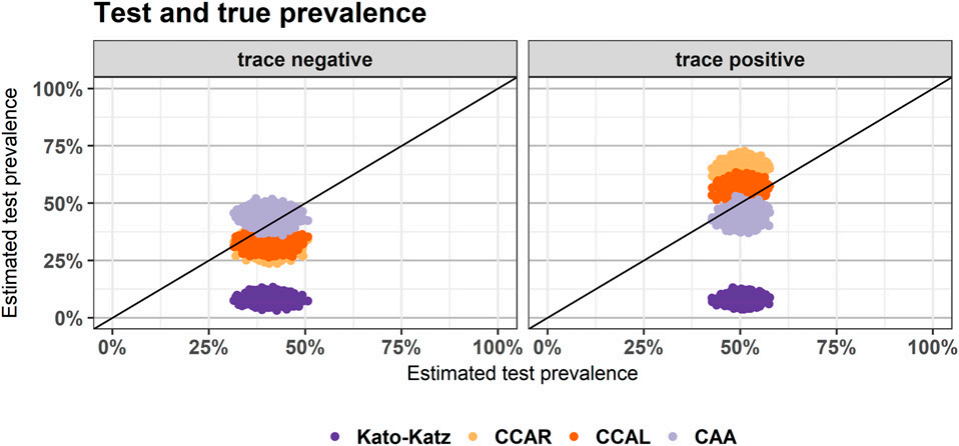
Estimated test prevalence against estimated disease prevalence when circulating cathodic antigen trace was considered negative (left) and positive (right). Estimates are from the output of a Bayesian latent class analysis and the cloud of dots for each test show 1,000 estimates from the posterior distribution.

Estimated infection prevalence in the eight schools was 40.7% (BCI: 35.8–45.8%; Table 4, Figure 4) when trace was considered negative and 50.6% (BCI: 46.7–54.5%) when trace was considered positive. Kato–Katz underestimated this prevalence significantly in both models: by 32.6% (BCI: 27.1–38.3%) when trace was considered negative and by 42.5% (BCI: 37.8–47.2%) when trace was considered positive. Point-of-care circulating cathodic antigen in Rwanda underestimated prevalence by 9.4% (BCI: 16.1–2.7%) when trace was considered negative and overestimated by 15.1% (BCI: 9.6–20.6%) when trace was considered positive.

The model with trace considered negative estimated that 68.3% (BCI: 58.0–78.5%) of all individuals who tested positive by POC-CCA in Rwanda were truly positive, and the model with trace considered positive estimated that 64.5% (BCI: 59.9–68.9%) individuals who tested positive were truly positive. Combining these results suggest that 61.1% (BCI: 50.1%, 72.1%) of all trace readings were from truly infected individuals.

## DISCUSSION

We presented results from the 2014 remapping of Rwanda in 388 schools for *S. mansoni*, following repeated rounds of treatment in higher prevalence areas since the program was established in 2007.^16,22^ As mapping was conducted using a new field technique, POC-CCA, we also reported comparison with the standard field technique, KK, in 175 schools that were tested using both techniques. In addition, we investigated the high proportion of trace results found by POC-CCA in Rwanda by retesting samples in Leiden from eight selected schools with POC-CCA and then also a newer laboratory-based method, UCP-LF CAA. We discuss here the main findings and implications to the control program.

This survey used mapping units rather than districts which were based on risk factors such as distance from major perennial waterbodies. Point-of-care circulating cathodic antigen was used as the diagnostic method for this mapping survey as it is considered more sensitive, especially in settings of low intensity and low prevalence.^26,34^ Similar to studies in other countries with long-standing MDA programs such as Burundi,^11^ Cameroon,^35^ and Malawi,^36^ this survey has demonstrated current endemicity of *S. mansoni* in Rwanda and highlighted areas in most need of treatment. All mapping units showed ongoing infections by CCA trace negative and prevalence above 10% by CCA trace positive. Mapping unit prevalence of at least 10% by CCA trace negative was observed in six of 21 high-risk mapping units and zero of 10 low-risk mapping units, whereas only two high-risk mapping units had prevalence above 50% by CCA trace positive, although eight high-risk mapping units and two low-risk mapping units had prevalence above 40% by CCA trace positive.

Although the organization of the mapping units in this survey was based on risk factors, prevalence at the school level still varied significantly within mapping units which confirmed the focal transmission of *S. mansoni*.^11,22,36^ The only three schools where no infections or trace readings were registered were in mapping units with substantial overall prevalences and maximum school prevalences up to 96% (mapping units 6, 16, and 21; see Supplemental Table S3). Indeed, in mapping unit 6, a quarter of all schools had a POC-CCA trace-positive prevalence of below 10%. In these mapping units, a more focal approach might be warranted.

Findings from the 175 schools surveyed with KK and POC-CCA found higher prevalence of infection by POC-CCA than by duplicate KK, reflecting other results from diverse geographic and schistosomiasis-endemic settings.^7,11^ Positive cases determined by KK were few and most infections were of light intensity.^22^ All mapping units which had a prevalence of 0% with KK still had positive results by POC-CCA. The minimum, mean, and maximum school prevalence measured by POC-CCA at KK-positive and KK-negative schools was similar to the values reported in a similar study in Burundi,^11^ also supported by the SCORE project. However, KK-negative schools were identified as endemic based predominantly on trace readings. In addition, the proportion of POC-CCA trace readings was higher in KK-negative pupils by a factor 1.6 than in KK-positive pupils, indicating that trace readings play an important part in identifying endemic schools in low-prevalence areas. These findings confirm the limitations of the KK technique to inform the control and progress toward elimination for national programs, especially in a context, like Rwanda, where the prevalence is generally low to moderate.^22^

The comparison of the diagnostic tools by the LCA showed that the sensitivity of KK was very low compared with the other tools. Whereas the estimate of the KK sensitivity outputted by the trace-negative model was similar to the results obtained by the Burundi study, the estimate of 2.5% from the trace positive model was much lower than the result in Burundi. This might be due to the higher proportion of trace readings in the Rwanda data compared with Burundi. However, both sets of results are consistent in underlining the insufficiency of the sensitivity of single stool KK in low-prevalence areas.

The sensitivity of POC-CCA detection in Rwanda when trace was considered negative was more than three times the sensitivity of KK and its specificity was only slightly lower than that of KK. The corresponding values from the study in Burundi were around 15% higher.^12^ When trace was considered, positive sensitivity of POC-CCA in Rwanda increased at the expense of the specificity. Our PPV estimates indicated that at least 50% of all traces in Rwanda were true infections, that is, with 34.4% trace readings in the eight Leiden schools in Rwanda, 17% or more true infections were missed when trace was considered negative. Other studies suggest slightly lower or even much higher percentages.^12,37^ Sensitivity and specificity estimates for POC-CCA in Leiden were among the highest above 90%, only specificity for the trace negative model was 86%. Unfortunately, sensitivity and specificity changed in the wrong direction with the transition from trace considered negative to trace considered positive. This is also reflected by the bad fit of both models for the POC-CCA Leiden data.

A strength of this study is the inclusion of CAA in the comparison of the diagnostic tools. Other studies have found a high sensitivity and specificity of CAA,^12,25^ which was at least partially confirmed by this study. The trace-negative model indicated a near 100% sensitivity and a high specificity of 83%. The trace-positive model showed, especially for the specificity, worse values; however, this model fitted the UCP-LF CAA prevalences unsatisfactorily, and these results, therefore, should be regarded with caution. If we assume all CAA positives were truly positive, then almost 90% of those were detected as positive with POC-CCA in Rwanda and in Leiden when trace was considered positive. With trace considered negative, only 52% were identified as positive by POC-CCA in Rwanda.

For this mapping study in Rwanda, almost 30% of all POC-CCA readings were trace results, and the same percentage of schools were identified as endemic based on trace only (Supplemental Table S6). Assuming approximately 50% of traces were true positives, around 15% of infections would have been missed by considering traces as negative. Even if individuals with trace readings have lower intensities of infection than individuals with higher POC-CCA readings as suggested elsewhere,^38^ this is still a considerable proportion which could be enough to keep endemicity alive.^39^ In the eight schools with samples tested in Leiden, 34.4% of POC-CCA results were trace in Rwanda and 21.96% were trace in Leiden. Although the proportion of trace results were still substantial when tested in Leiden, the additional traces when tested in Rwanda could reflect the influence of subjectivity in test result interpretation and also the influence of external factors, such as light conditions, on the reading.^40^

This study has several limitations. First, the mapping was school-based, and consequently, we did not test children who did not attend school. Attendance rates in Rwanda is relatively high (approximately 70%), and so, we hope that prevalence among school attendees, coupled with the large number of schools sampled (almost 400), accurately reflects prevalence in the wider population. In addition, the risk zones constructed did not seem to accurately reflect prevalence as tested. The risk zones used proximity to water as a stratifying factor, although data on man-made wetlands and water dams, which may play an important role in transmission,^41^ were not available. Other factors such as local sanitation, biological transmission factors,^42^ or number of previous rounds of MDA may also contribute to the prevalence observed. Finally, although we used a number of different tests, no test is perfect, with each differing in sensitivity and specificity, and the use of multiple tests can complicate interpretation. Indeed, although the prevalence estimates by POC-CCA trace negative were similar when measured in Rwanda and Leiden, only 60% of samples recorded the same result in each test, with the vast majority of the remaining samples differing by just one level. It is not clear whether this result reflects true biological differences (e.g., changes in the sample from storage and shipping) or processing differences (e.g., inter-reader differences), but this is an area of active research within the scientific community.^34^ We also used LCA to better understand the differences observed in the test results and noted much higher sensitivity of both POC-CCA and CAA than KK.

This study adds a valuable contribution to the literature on the use of POC-CCA as mapping tool in low-intensity areas. In addition, it provides Rwandan public health officials with critical information on where and how to pursue control and eventual elimination of schistosomiasis. The large number of mapping units and their definition linked to transmission characteristics allowed a more accurate estimate of the prevalence and provided an opportunity to study the link between prevalence levels and proximity to perennial water bodies.

The objective of this mapping study was to inform the government on future treatment strategies in Rwanda. It is clear from these results that mapping with KK alone would have been insufficient. Whereas in more than half of the mapping units, the KK failed to detect infections, POC-CCA with trace negative detected ongoing infections in all mapping units. Whereas only six mapping units had a prevalence above 10% when trace was considered negative, this was the case for all mapping units for trace taken as positive, which would require even higher treatment efforts following the WHO guidelines. This not only demonstrated that single stool duplicate slide of the KK method dramatically underestimates *S. mansoni* infection prevalence but also demonstrates the importance of interpretation of POC-CCA trace readings in low-intensity areas. The choice to use these different assays and various survey protocols will remain programmatic decisions and will likely vary depending on the goals and targets set by a country’s NTD program, as well as those recommended by new WHO guidelines for schistosomiasis control and elimination that are currently being deliberated.

## Supplemental tables

Supplemental materials

## References

[1] Vos T 2015 Global, regional, and national incidence, prevalence, and years lived with disability for 301 acute and chronic diseases band injuries in 188 countries, 1990–2013: a systematic analysis for the Global Burden of Disease Study 2013. Lancet 386: 743–800. 26063472 10.1016/S0140-6736(15)60692-4 PMC4561509

[2] Hotez PJ Kamath A, 2009 Neglected tropical diseases in sub-Saharan Africa: review of their prevalence, distribution, and disease burden. PLoS Negl Trop Dis 3: e412. 19707588 10.1371/journal.pntd.0000412 PMC2727001

[3] Steinmann P Keiser J Bos R Tanner M Utzinger J, 2006 Schistosomiasis and water resources development: systematic review, meta-analysis, and estimates of people at risk. Lancet Infect Dis 6: 411–425. 16790382 10.1016/S1473-3099(06)70521-7

[4] WHO, 2011 Helminth Control in School-Age Children, 2nd edition Geneva, Switzerland: World Health Organization.

[5] Katz N Chaves A Pellegrino J, 1972 A simple device for quantitative stool thick smear technique in *Schistosomiasis mansoni*. Rev Soc Bras Med Trop 14: 397–400. 4675644

[6] WHO, 2006 Preventive Chemotherapy in Human Helminthiasis. Geneva, Switzerland: World Health Organization.

[7] Colley DG 2013 A five-country evaluation of a point-of-care circulating cathodic antigen urine assay for the prevalence of *Schistosoma mansoni*. Am J Trop Med Hyg 88: 426–432. 23339198 10.4269/ajtmh.12-0639 PMC3592520

[8] Lamberton PHL Kabatereine NB Oguttu DW Fenwick A Webster JP, 2014 Sensitivity and specificity of multiple kato-katz thick smears and a circulating cathodic antigen test for *Schistosoma mansoni* diagnosis pre- and post-repeated-praziquantel treatment. PLoS Negl Trop Dis 8: e3139. 25211217 10.1371/journal.pntd.0003139 PMC4161328

[9] Kittur N Castleman JD Campbell CH King CH Colley DG, 2016 Comparison of *Schistosoma mansoni* prevalence and intensity of infection, as determined by the circulating cathodic antigen urine assay or by the kato-katz fecal assay: a systematic review. Am J Trop Med Hyg 94: 605–610. 26755565 10.4269/ajtmh.15-0725 PMC4775897

[10] WHO, 2012 Accelerating Work to Overcome the Global Impact of Neglected Tropical Diseases. A Roadmap for Implementation. Geneva, Switzerland: World Health Organisation.

[11] Ortu G 2017 Countrywide reassessment of *Schistosoma mansoni* infection in Burundi using a urine-circulating cathodic antigen rapid test: informing the national control program. Am J Trop Med Hyg 96: 664–673. 28115675 10.4269/ajtmh.16-0671 PMC5361543

[12] Clements MN 2018 Latent class analysis to evaluate performance of point-of-care CCA for low-intensity *Schistosoma mansoni* infections in Burundi. Parasit Vectors 11: 111. 29475457 10.1186/s13071-018-2700-4 PMC5824563

[13] Corstjens PLAM Van Lieshout L Zuiderwijk M Kornelis D Tanke HJ Deelder AM Van Dam GJ, 2008 Up-converting phosphor technology-based lateral flow assay for detection of *Schistosoma* circulating anodic antigen in serum. J Clin Microbiol 46: 171–176. 17942645 10.1128/JCM.00877-07 PMC2224263

[14] Corstjens PLAM Nyakundi RK De Dood CJ Kariuki TM Ochola EA Karanja DMS Mwinzi PNM Van Dam GJ, 2015 Improved sensitivity of the urine CAA lateral-flow assay for diagnosing active *Schistosoma* infections by using larger sample volumes. Parasit Vectors 8: 241. 25896512 10.1186/s13071-015-0857-7 PMC4418045

[15] Mupfasoni D 2009 Polyparasite helminth infections and their association to anaemia and undernutrition in northern Rwanda. PLoS Negl Trop Dis 3: e517. 19753110 10.1371/journal.pntd.0000517 PMC2737105

[b16] Ruxin J Negin J, 2012 Removing the neglect from neglected tropical diseases: the Rwandan experience 2008–2010. Glob Public Health 7: 812–822. 22812700 10.1080/17441692.2012.699535

[17] Ruberanziza E 2010 A recent update of Schistomiasis mansoni endemicity around lake Rweru. Neglected tropical disease (NTD) control programme, The Access Project. Rwanda Med J 68.

[b18] Ruberanziza E 2015, Nkombo island: the most important *Schistosomiasis mansoni* focus in Rwanda. Am J Life Sci 3: 27.

[19] Rwanda Development Board, 2017 About Rwanda. Available at: http://rdb.rw/about-rwanda/#geography. Accessed December 28, 2017.

[20] National Institute of Statistics of Rwanda, 2014 Fourth Population and Housing Census, Rwanda, 2012. CENSUS ATLAS Available at: http://www.statistics.gov.rw/publication/rphc4-atlas.

[21] National Institute of Statistics of Rwanda, 2014 Socio-Economic Status of Children. Kigali, Rwanda Available at: http://www.statistics.gov.rw/publication/rphc4-thematic-report-socio-economic-status-children.

[22] Rujeni N Morona D Ruberanziza E Mazigo HD, 2017 Schistosomiasis and soil-transmitted helminthiasis in Rwanda: an update on their epidemiology and control. Infect Dis Poverty 6: 1–11. 28245883 10.1186/s40249-016-0212-z PMC5331630

[23] Naing L Winn T Rusli BN, 2006 Practical issues in calculating the sample size for prevalence studies. Arch Orofacial Sci 1: 9–14.

[24] Ruberanziza E 2010, A recent update of schistosomiasis mansoni endemicity around Lake Rweru. Rwanda Med J 68: 5–9.

[25] Stothard JR Kabatereine NB Tukahebwa EM Kazibwe F Rollinson D Mathieson W Webster JP Fenwick A, 2006 Use of circulating cathodic antigen (CCA) dipsticks for detection of intestinal and urinary schistosomiasis. Acta Trop 97: 219–228. 16386231 10.1016/j.actatropica.2005.11.004

[26] Corstjens PLAM 2014 Tools for diagnosis, monitoring and screening of *Schistosoma* infections utilizing lateral-flow based assays and upconverting phosphor labels. Parasitology 141: 1841–1855. 24932595 10.1017/S0031182014000626 PMC4265670

[27] Team RC, 2017 R: A Language and Environment for Statistical Computing. Available at: https://www.r-project.org/.

[28] Bates D Mächler M Bolker B Walker S, 2015 Fitting linear mixed-effects models using lme4. J Stat Soft 67: 1–48.

[29] Skaug H Fournier DNA, 2016 glmmADMB: Generalized Linear Mixed Models Using AD Model Builder.

[30] Dendukuri N Joseph L, 2001 Bayesian approaches to modeling the conditional dependence between multiple diagnostic tests. Biometrics 57: 158–167. 11252592 10.1111/j.0006-341x.2001.00158.x

[31] Lunn D Spiegelhalter D Thomas A Best N, 2009 The BUGS project: evolution, critique and future directions. Stat Med 28: 3049–3067. 19630097 10.1002/sim.3680

[32] Stevenson M 2019 epiR: Tools for the Analysis of Epidemiological Data. R Package Version 1.0-2. Available at: https://cran.r-project.org/package=epiR.

[33] Brooks SP Gelman A, 1997 General methods for monitoring convergence of iterative simulations. J Comput Graph Stat 7: 434–455.

[34] Mwinzi PNM Kittur N Ochola E Cooper PJ Campbell CH King CH Colley DG, 2015 Additional evaluation of the point-of-contact circulating cathodic antigen assay for *Schistosoma mansoni* infection. Front Public Heal 3: 48. 10.3389/fpubh.2015.00048 PMC4365547 25853117

[35] Tchuem Tchuenté L-A 2012 Mapping of schistosomiasis and soil-transmitted helminthiasis in the regions of centre, east and west Cameroon. PLoS Negl Trop Dis 6: e1553. 22413029 10.1371/journal.pntd.0001553 PMC3295801

[36] Makaula P Sadalaki JR Muula AS Kayuni S Jemu S Bloch P, 2014 Schistosomiasis in Malawi: a systematic review. Parasit Vectors 7: 570. 25490938 10.1186/s13071-014-0570-y PMC4288699

[37] Prada JM Touloupou P Adriko M Tukahebwa EM Lamberton PHL Hollingsworth TD, 2017 Understanding the relationship between egg-and antigen-based diagnostics of *Schistosoma mansoni* infection pre-and post-treatment in Uganda. Parasit Vectors 11: 21. 10.1186/s13071-017-2580-z PMC5759883 29310695

[38] Haggag AA Rabiee A Abd Elaziz KM Campbell CH Colley DG Ramzy RMR, 2019 Thirty-day daily comparisons of Kato–Katz and CCA assays of 45 Egyptian children in areas with very low prevalence of *Schistosoma mansoni*. Am J Trop Med Hyg 100: 578–583. 30608053 10.4269/ajtmh.18-0829 PMC6402931

[39] Evan Secor W, 2014 Water-based interventions for schistosomiasis control. Pathog Glob Health 108: 246–254. 25175875 10.1179/2047773214Y.0000000149 PMC4153826

[40] Viana AG 2019 Discrepancy between batches and impact on the sensitivity of point-of-care circulating cathodic antigen tests for *Schistosoma mansoni* infection. Acta Trop 197: 105049. 31158344 10.1016/j.actatropica.2019.105049

[41] Appleton CC Madsen H, 2012 Human schistosomiasis in wetlands in southern Africa. Wetl Ecol Manag 20: 253–269.

[42] Clark NJ 2019 Mapping *Schistosoma mansoni* endemicity in Rwanda: a critical assessment of geographical disparities arising from circulating cathodic antigen versus kato-katz diagnostics. PLoS Negl Trop Dis 13: e0007723. 31568504 10.1371/journal.pntd.0007723 PMC6786642

