# Supplementary material for: Nationwide Remapping of Schistosoma mansoni Infection in Rwanda Using Circulating Cathodic Antigen Rapid Test: Taking Steps toward Elimination
Source: Am J Trop Med Hyg. 2020 May 18;103(1):315–24. doi: 10.4269/ajtmh.19-0866 (PMC7356434; doi:10.4269/ajtmh.19-0866)
Supplement: Supplementary file 1 [file tpmd190866.SD1.docx]

# Supplemental MAterials

| **Parameter** | **Test** | **α** | **β** | **95% greater than** | **Mode** | **Sample size equivalent** |
| --- | --- | --- | --- | --- | --- | --- |
| Sensitivity | KK | 1.43 | 1.29 | 10% | 60% | 2.72 |
| Specificity | KK | 21.2 | 2.06 | 80% | 95% | 23.26 |
| Sensitivity | CCAB CCAL CAA | 3.05 | 1.51 | 30% | 80% | 4.56 |
| Specificity | CCAB CCAL CAA | 5.38 | 1.49 | 50% | 90% | 6.87 |

Table S1: Priors used in Bayesian Latent Class analysis. Alpha and Beta are the parameters of the Beta distribution with associated properties of 95% greater than and mode. The sum of alpha and beta is often known as the ‘sample size equivalent’ and the effect of the prior is equivalent to adding alpha + beta samples to the analysis, with alpha samples being positive.

| **Covariance** | **DIC Trace negative** | **DIC Trace positive** |
| --- | --- | --- |
| no covariance | 300.4 | 412.8 |
| KK CCA Rwanda | 304 | 376.7 |
| KK CCAL | 305 | 391.9 |
| KK & CAA | 242.4 | **286.3 (*)** |
| CCAR & CCAL | 213.6 | 403 |
| CCAR & CAA | 304.4 | 414.3 |
| CCA L & CAA | 305.3 | didn't converge |
| KK & CAA and CCAR & CCAL | **153.7** | 283.5 |

Table S2: The Deviance Information Criteria (DIC) used for the selection of the Latent Class Analysis model. The first column lists the combination of covariance terms incorporated in the model, the second and third column lists the resulting DIC values for the trace positive and trace negative model respectively. As differences between DIC values of less than five can be misleading the simpler of the two models with the lowest DIC was used when trace was considered positive.

| **Mapping unit** | **Mapping unit risk zone** | **Number of schools** | **Number of pupils** | **Prevalence by CCA trace negative (Minimum, Q25, Q75, maximum on schools level)** | **Prevalence by CCA trace positive (Minimum, Q25, Q75, maximum on school level)** |
| --- | --- | --- | --- | --- | --- |
| 1 | H | 14 | 700 | 15.4% (0%, 2%, 17%, 90%) | 35.7% (4%, 22%, 44%, 98%) |
| 2 | H | 18 | 899 | 16.7% (0%, 1%, 27%, 74%) | 42.9% (10%, 17%, 72%, 98%) |
| 3 | H | 16 | 800 | 7.1% (0%, 0%, 8%, 34%) | 28.5% (10%, 14%, 35%, 68%) |
| 4 | H | 16 | 797 | 16.4% (0%, 2%, 22%, 90%) | 41.4% (14%, 26%, 63%, 100%) |
| 5 | H | 13 | 650 | 9.2% (0%, 4%, 10%, 30%) | 63.5% (34%, 50%, 74%, 94%) |
| 6 | H | 23 | 1147 | 7.5% (0%, 0%, 6%, 48%) | 37.8% (0%, 9%, 64%, 96%) |
| 7 | H | 14 | 691 | 16.2% (0%, 6%, 18%, 74%) | 53.7% (29%, 39%, 66%, 96%) |
| 8 | H | 14 | 700 | 15.3%  (0%, 3%, 16%, 80%) | 47.4% (12%, 29%, 63%, 98%) |
| 9 | H | 13 | 650 | 0.6% (0%, 0%, 0%, 4%) | 22.9% (10%, 16%, 28%, 34%) |
| 10 | H | 13 | 649 | 3.4% (0%, 0%, 4%, 10%) | 25% (6%, 18%, 32%, 44%) |
| 11 | H | 13 | 650 | 8.6% (0%, 2%, 14%, 20%) | 32.6% (14%, 20%, 42%, 58%) |
| 12 | H | 13 | 650 | 2.6% (0%, 0%, 6%, 6%) | 28.6% (6%, 24%, 34%, 56%) |
| 13 | H | 14 | 696 | 9.5% (0%, 4%, 10%, 46%) | 43.5% (11%, 34%, 48%, 86%) |
| 14 | H | 14 | 699 | 6.9% (0%, 0%, 4%, 45%) | 37.9% (20%, 29%, 42%, 76%) |
| 15 | H | 13 | 650 | 2.8% (0%, 0%, 4%, 8%) | 31.8% (10%, 18%, 40%, 64%) |
| 16 | H | 17 | 850 | 8.8% (0%, 4%, 14%, 22%) | 40.1% (0%, 28%, 48%, 74%) |
| 17 | H | 15 | 750 | 8.3% (0%, 0%, 12%, 36%) | 40.9% (8%, 27%, 56%, 74%) |
| 18 | H | 12 | 598 | 2.2% (0%, 0%, 4%, 6%) | 25.1% (4%, 13%, 38%, 52%) |
| 19 | H | 14 | 700 | 10.6% (0%, 3%, 15%, 34%) | 39.6% (4%, 25%, 61%, 72%) |
| 20 | L | 11 | 550 | 4.5% (0%, 3%, 6%, 10%) | 28.9% (8%, 22%, 34%, 60%) |
| 21 | L | 9 | 450 | 6.2% (0%, 0%, 6%, 24%) | 48.2% (0%, 34%, 56%, 92%) |
| 22 | L | 9 | 450 | 2.4% (0%, 0%, 2%, 12%) | 47.6% (30%, 40%, 52%, 72%) |
| 23 | L | 9 | 450 | 4.7% (0%, 2%, 4%, 16%) | 37.3% (22%, 32%, 46%, 50%) |
| 24 | H | 4 | 200 | 4.5% (2%, 2%, 7%, 8%) | 36.5% (26%, 37%, 40%, 40%) |
| 25 | L | 9 | 450 | 3.1% (0%, 0%, 6%, 8%) | 23.6% (10%, 12%, 26%, 48%) |
| 26 | L | 9 | 450 | 1.1% (0%, 0%, 2%, 6%) | 22.7% (10%, 14%, 32%, 44%) |
| 27 | L | 9 | 445 | 1.6% (0%, 0%, 2%, 4%) | 25.4% (6%, 16%, 32%, 48%) |
| 28 | L | 9 | 450 | 3.1% (0%, 2%, 4%, 8%) | 35.1% (22%, 32%, 38%, 46%) |
| 29 | L | 9 | 450 | 3.8% (0%, 2%, 6%, 8%) | 37.1% (16%, 30%, 48%, 62%) |
| 30 | L | 9 | 450 | 2.9% (0%, 0%, 6%, 6%) | 33.6% (16%, 26%, 42%, 44%) |
| 31 | H | 13 | 650 | 0.6% (0%, 0%, 0%, 4%) | 10.8% (4%, 4%, 12%, 34%) |

Supplementary Table S3: Raw prevalence estimates in each mapping unit in Rwanda CCA mapping in 2014.

|  |  | R2.marginal | R2.conditional |  |
| --- | --- | --- | --- | --- |
| (Intercept) |  | 0.16 | 0.46 |  |
|  |  |  |  |  |
| Fixed Effects | Level | Parameter | adjOddsRatio | pValues |
| (Intercept) |  | -5.99 (0.68) | 0 (0, 0.01) | <0.001 |
| Sex | F |  |  |  |
|  | M | 0.31 (0.063) | 1.36 (1.2, 1.54) | <0.001 |
| Age.s |  | 0.04 (0.037) | 1.04 (0.97, 1.12) | 0.268 |
| I(Age.s^2) |  | -0.05 (0.023) | 0.95 (0.91, 0.99) | 0.027 |
| Mapping Unit | Unit 31 |  |  |  |
|  | Unit 1 | 3.33 (0.79) | 28.08 (5.97, 132.15) | <0.001 |
|  | Unit 2 | 3.38 (0.77) | 29.32 (6.52, 131.78) | <0.001 |
|  | Unit 3 | 2.45 (0.79) | 11.58 (2.47, 54.34) | 0.002 |
|  | Unit 4 | 3.54 (0.77) | 34.4 (7.54, 156.94) | <0.001 |
|  | Unit 5 | 3.07 (0.8) | 21.58 (4.52, 103.06) | <0.001 |
|  | Unit 6 | 2.36 (0.76) | 10.56 (2.38, 46.88) | 0.002 |
|  | Unit 7 | 3.82 (0.78) | 45.62 (9.84, 211.53) | <0.001 |
|  | Unit 8 | 3.53 (0.79) | 34.16 (7.32, 159.33) | <0.001 |
|  | Unit 9 | 0.01 (0.96) | 1.01 (0.15, 6.59) | 0.996 |
|  | Unit 10 | 1.88 (0.83) | 6.54 (1.3, 33.03) | 0.023 |
|  | Unit 11 | 3.1 (0.8) | 22.2 (4.66, 105.71) | <0.001 |
|  | Unit 12 | 1.86 (0.83) | 6.45 (1.26, 33.12) | 0.026 |
|  | Unit 13 | 3.14 (0.79) | 23.17 (4.94, 108.63) | <0.001 |
|  | Unit 14 | 1.94 (0.82) | 6.98 (1.4, 34.89) | 0.018 |
|  | Unit 15 | 1.75 (0.83) | 5.77 (1.14, 29.34) | 0.034 |
|  | Unit 16 | 3.12 (0.77) | 22.74 (5.03, 102.87) | <0.001 |
|  | Unit 17 | 2.65 (0.79) | 14.09 (2.99, 66.53) | 0.001 |
|  | Unit 18 | 1.5 (0.85) | 4.5 (0.84, 23.92) | 0.078 |
|  | Unit 19 | 3.28 (0.79) | 26.51 (5.67, 124.03) | <0.001 |
|  | Unit 20 | 2.49 (0.83) | 12.07 (2.38, 61.13) | 0.003 |
|  | Unit 21 | 2.41 (0.86) | 11.18 (2.05, 60.87) | 0.005 |
|  | Unit 22 | 1.35 (0.92) | 3.85 (0.63, 23.37) | 0.143 |
|  | Unit 23 | 2.32 (0.86) | 10.22 (1.88, 55.68) | 0.007 |
|  | Unit 24 | 2.5 (1) | 12.14 (1.6, 92.03) | 0.016 |
|  | Unit 25 | 1.91 (0.88) | 6.78 (1.2, 38.26) | 0.03 |
|  | Unit 26 | 0.6 (0.98) | 1.82 (0.27, 12.38) | 0.542 |
|  | Unit 27 | 1.19 (0.93) | 3.29 (0.54, 20.28) | 0.198 |
|  | Unit 28 | 1.99 (0.88) | 7.33 (1.32, 40.84) | 0.023 |
|  | Unit 29 | 2.23 (0.87) | 9.29 (1.7, 50.74) | 0.01 |
|  | Unit 30 | 1.85 (0.88) | 6.36 (1.13, 35.88) | 0.036 |
| Random Effects | | Var | Sd |  |
| School (Intercept) | | 1.8 | 1.3 |  |

*Table S4: Output from Binomial mixed model analysis of prevalence by CCA trace negative. Fixed effects were age, sex and mapping unit, with school being the only random effect. Mapping unit 31 with the lowest prevalence was taken as reference mapping unit.*

|  |  | R2.marginal | R2.conditional |  |
| --- | --- | --- | --- | --- |
| (Intercept) |  | 0.069 | 0.2625 |  |
|  |  |  |  |  |
| Fixed Effects | Level | Parameter | adjOddsRatio | pValues |
| (Intercept) |  | -2.39 (0.3) | 0.09 (0.05, 0.16) | <0.001 |
| Sex | F |  |  |  |
|  | M | 0.08 (0.033) | 1.09 (1.02, 1.16) | 0.013 |
| Age.s |  | -0.03 (0.02) | 0.97 (0.93, 1.01) | 0.135 |
| I(Age.s^2) |  | -0.01 (0.01) | 0.99 (0.97, 1.01) | 0.413 |
| Mapping Unit | Unit 31 |  |  |  |
|  | Unit 1 | 1.71 (0.4) | 5.52 (2.53, 12.02) | <0.001 |
|  | Unit 2 | 2.05 (0.38) | 7.73 (3.69, 16.19) | <0.001 |
|  | Unit 3 | 1.31 (0.39) | 3.7 (1.74, 7.89) | 0.001 |
|  | Unit 4 | 2 (0.38) | 7.4 (3.48, 15.74) | <0.001 |
|  | Unit 5 | 3 (0.4) | 20.14 (9.16, 44.31) | <0.001 |
|  | Unit 6 | 1.64 (0.36) | 5.17 (2.53, 10.54) | <0.001 |
|  | Unit 7 | 2.55 (0.4) | 12.81 (5.9, 27.79) | <0.001 |
|  | Unit 8 | 2.26 (0.4) | 9.6 (4.42, 20.86) | <0.001 |
|  | Unit 9 | 1.07 (0.4) | 2.91 (1.32, 6.41) | 0.008 |
|  | Unit 10 | 1.15 (0.4) | 3.15 (1.43, 6.95) | 0.005 |
|  | Unit 11 | 1.57 (0.4) | 4.79 (2.18, 10.53) | <0.001 |
|  | Unit 12 | 1.33 (0.4) | 3.78 (1.71, 8.35) | 0.001 |
|  | Unit 13 | 2.05 (0.39) | 7.79 (3.59, 16.9) | <0.001 |
|  | Unit 14 | 1.83 (0.39) | 6.24 (2.88, 13.53) | <0.001 |
|  | Unit 15 | 1.5 (0.4) | 4.47 (2.03, 9.83) | <0.001 |
|  | Unit 16 | 1.85 (0.38) | 6.38 (3.03, 13.42) | <0.001 |
|  | Unit 17 | 1.92 (0.39) | 6.82 (3.18, 14.62) | <0.001 |
|  | Unit 18 | 1.08 (0.41) | 2.94 (1.31, 6.6) | 0.009 |
|  | Unit 19 | 1.83 (0.4) | 6.26 (2.88, 13.59) | <0.001 |
|  | Unit 20 | 1.35 (0.42) | 3.84 (1.69, 8.75) | 0.001 |
|  | Unit 21 | 2.22 (0.44) | 9.18 (3.85, 21.88) | <0.001 |
|  | Unit 22 | 2.28 (0.44) | 9.75 (4.12, 23.08) | <0.001 |
|  | Unit 23 | 1.8 (0.44) | 6.08 (2.57, 14.37) | <0.001 |
|  | Unit 24 | 1.79 (0.57) | 5.98 (1.96, 18.26) | 0.002 |
|  | Unit 25 | 1.05 (0.44) | 2.85 (1.19, 6.83) | 0.018 |
|  | Unit 26 | 1 (0.45) | 2.72 (1.14, 6.5) | 0.025 |
|  | Unit 27 | 1.11 (0.45) | 3.04 (1.27, 7.28) | 0.012 |
|  | Unit 28 | 1.71 (0.44) | 5.55 (2.35, 13.13) | <0.001 |
|  | Unit 29 | 1.76 (0.44) | 5.84 (2.46, 13.84) | <0.001 |
|  | Unit 30 | 1.62 (0.44) | 5.08 (2.14, 12.03) | <0.001 |
| Random Effects: | | Var | sd |  |
| School (Intercept) | | 0.86 | 0.93 |  |

*Table S5: Output from Binomial mixed model analysis of prevalence by CCA trace positive. Fixed effects were age, sex and mapping unit, with school being the only random effect. Mapping unit 31 with the lowest prevalence was taken as reference mapping unit.*

| Survey | **CCA mapping** | **KK schools** |
| --- | --- | --- |
| Number of mapping units | 31 | 31 |
| Number of schools | 388 | 175 |
| Number of pupils | 19371 | 8697 |
| Number (%) of girls | 9697 (50.1%) | 4337 (49.9%) |
| Mean age (SD) | 13.4 (0.75) | 13.3 (0.75) |
| Number CCA 0/trace/+/++/+++ | 12371/5566/880/291/263 | 5438/2513/479/147/120 |
| Percentage CCA 0/trace/+/++/+++ | 63.9/28.7/4.5/1.5/1.4 % | 62.5/28.9/5.5/1.7/1.4 % |
| Percentage of children with infection: CCA trace negative (school IQR) | 7.4% (0.0 - 8.0) | 8.6% (1.0 - 8.0) |
| Percentage of children with infection: CCA trace positive (school IQR) | 36.1% (20.0 - 46.5) | 37.5% (22.0 - 49.0) |
| Percentage of schools with infection: CCA trace negative | 70.6% | 74.9% |
| Percentage of schools with infection: CCA trace positive | 99.2% | 98.9% |
| Number of KK uninfected / light / moderate / heavy infections |  | 8525/129/39/4 |
| Percentage of KK uninfected/light/moderate/heavy infections |  | 98.0/1.5/0.4/0.0% |
| Percentage of children with infection: KK (school IQR) |  | 2.0% (0.0 - 0.0) |
| Percentage of schools with infection: KK |  | 18.9% |

Table S6**:**Summary statistics and prevalence by CCA and KK from 388 CCA mappings schools and from 175 KK schools.

| **KK** | **CCA** | **Number of pupils** | **Proportion of pupils** |
| --- | --- | --- | --- |
| KK negative | 0 | 5425 | 63.6% |
|  | 1 | 2482 | 29.1% |
|  | 2 | 454 | 5.3% |
|  | 3 | 111 | 1.3% |
|  | 4 | 53 | 0.6% |
| KK positive | 0 | 13 | 7.6% |
|  | 1 | 31 | 18.0% |
|  | 2 | 25 | 14.5% |
|  | 3 | 36 | 20.9% |
|  | 4 | 67 | 39.0% |

Table S7. CCA results from 175 KK and CCA schools broken up in pupils that were KK positive and pupils that were KK negative

|  |  | KK | |
| --- | --- | --- | --- |
|  | CCA reading  (N) | Negative Percentage (N) | Positive Percentage (N) |
| CCA (N) | 0 (5438) | 99.76%  (5425) | 0.24%  (13) |
|  | 1(2513) | 98.77%  (2482) | 1.23%  (31) |
|  | 2(479) | 94.78%  (454) | 5.22%  (25) |
|  | 3(147) | 75.51%  (111) | 24.49%  (36) |
|  | 4(120) | 44.17%  (53) | 55.83%  (67) |

Table S8: CCA and KK results from 175 KK and CCA schools. Percentages in one row add up to 100%

| **Parameter** | **Eight schools assayed in Leiden** |
| --- | --- |
| SCH mapping unit | 3, 4, 6, 8, 17 |
| Number of Schools | 8 |
| Number of pupils | 396 |
| Number of girls / boys | 197 / 199 |
| Proportion of girls | 49.70% |
| Mean age (SD) | 13.44 (0.67) |
| Minimum age / Maximum age | 11/16 |
| Number of missing age | 0 |
| **CCA Results** | |
| Rwanda 0 / 1 / 2 / 3 / 4 / 5 | 136 / 136 / 64 / 30 / 30  (34%, 34%, 16%, 8%, 8%) |
| Leiden 0 / 1 / 2 / 3 / 4 / 5 | 175 / 87 / 77 / 41 / 16  (44%, 22%, 19%, 10%, 4%) |
| **KK results** | |
| KK negative / light / moderate / heavy | 364 / 25 / 7 / 0  (92%, 6%, 2%, 0%) |
| Mean intensity of infection: KK (epg; SD) | 5.58 (27.27) |
| **Prevalence (school IQR)** | |
| KK | 8.1% (3.0% - 11.2%) |
| CCA trace negative in Rwanda | 31.3% (32.0% - 37.1%) |
| CCA trace positive in Rwanda | 65.7% (64.0% - 78.5%) |
| CCA trace negative in Leiden | 33.84% (25.5% - 47.8%) |
| CCA trace positive in Leiden | 55.8% (50.0% - 75.0%) |
| CAA (school IQR) | 44.2% (31.0% - 63.5%) |

*Table S9:. Summary statistics and prevalence by KK, POC-CCA and UCP-LF CAA in 8 purposively selected schools. The urine samples were sent to Leiden for additional testing by both POC-CCA and UCP-LF CAA assays. ‘Number’ and ‘percentage’*

|  | **CCARwanda** |  |  |  |  |  |
| --- | --- | --- | --- | --- | --- | --- |
| **CCA Leiden** | **negative** | **trace** | **+** | **++** | **+++** | **Total** |
| **negative** | 83.1% (113) | 41.2% (56) | 9.4% (6) | 0.0% (0) | 0.0% (0) | 44.2% (175) |
| **trace** | 14.0% (19) | 42.6% (58) | 15.6% (10) | 0.0% (0) | 0.0% (0) | 22.0% (87) |
| **+** | 2.2% (3) | 14.7% (20) | 59.4% (38) | 46.7% (14) | 6.7% (2) | 19.4% (77) |
| **++** | 0.7% (1) | 1.5% (2) | 15.6% (10) | 40.0% (12) | 53.3% (16) | 10.4% (41) |
| **+++** | 0.0% (0) | 0.0% (0) | 0.0% (0) | 13.3% (4) | 40.0% (12) | 4.0% (16) |
| Total | 100.0% (136) | 100.0% (136) | 100.0% (64) | 100.0% (30) | 100.0% (30) | 100.0% (396) |

Table S10: Comparison between CCA readings in Rwanda and Leiden for the sample from the 8 Leiden schools. Numbers in brackets are absolute counts.

| **CCA Trace Negative** | | |  | School | | | | | | | |  |
| --- | --- | --- | --- | --- | --- | --- | --- | --- | --- | --- | --- | --- |
| KK | CCA Rwanda | CCA Leiden | CAA | 1 | 2 | 3 | 4 | 5 | 6 | 7 | 8 | Total |
| 0 | 0 | 0 | 0 | 17 | 25 | 48 | 36 | 18 | 27 | 13 | 11 | 195 |
| 0 | 0 | 0 | 1 | 6 | 5 | 2 | 1 | 8 | 5 | 6 | 17 | 50 |
| 0 | 0 | 1 | 0 | 0 | 1 | 0 | 0 | 1 | 0 | 1 | 1 | 4 |
| 0 | 0 | 1 | 1 | 4 | 1 | 0 | 0 | 1 | 1 | 9 | 4 | 20 |
| 0 | 1 | 0 | 0 | 0 | 2 | 0 | 13 | 0 | 1 | 0 | 0 | 16 |
| 0 | 1 | 1 | 0 | 2 | 2 | 0 | 0 | 2 | 0 | 0 | 0 | 6 |
| 0 | 1 | 1 | 1 | 16 | 9 | 0 | 0 | 16 | 6 | 11 | 15 | 73 |
| 1 | 0 | 0 | 1 | 0 | 0 | 0 | 0 | 1 | 0 | 0 | 0 | 1 |
| 1 | 0 | 1 | 1 | 0 | 0 | 0 | 0 | 1 | 0 | 1 | 0 | 2 |
| 1 | 1 | 1 | 1 | 4 | 5 | 0 | 0 | 2 | 10 | 6 | 2 | 29 |

Table S11: Combinations of test results from the 8 schools purposively selected for further sample analysis in Leiden. 0 in the test results denotes negative and 1 denotes positive by the respective test. The number of children with each test combination is each school is shown on the right hand side. For example, 17 children in school 1 tested negative on all four tests when POC-CCA trace was negative (0-0-0-0 in top row)

| **CCA Trace Positive** | | | | School Code | | | | | | | |  |
| --- | --- | --- | --- | --- | --- | --- | --- | --- | --- | --- | --- | --- |
| KK | CCA Rwanda | CCA Leiden | CAA | 1 | 2 | 3 | 4 | 5 | 6 | 7 | 8 | Total |
| 0 | 0 | 0 | 0 | 10 | 10 | 44 | 4 | 1 | 19 | 8 | 7 | 103 |
| 0 | 0 | 0 | 1 | 2 | 1 | 2 | 0 | 0 | 0 | 1 | 4 | 10 |
| 0 | 0 | 1 | 0 | 1 | 1 | 4 | 0 | 1 | 2 | 2 | 1 | 12 |
| 0 | 0 | 1 | 1 | 0 | 1 | 0 | 0 | 0 | 0 | 5 | 5 | 11 |
| 0 | 1 | 0 | 0 | 4 | 6 | 0 | 35 | 7 | 1 | 0 | 0 | 53 |
| 0 | 1 | 0 | 1 | 1 | 1 | 0 | 1 | 2 | 0 | 1 | 2 | 8 |
| 0 | 1 | 1 | 0 | 4 | 13 | 0 | 10 | 12 | 6 | 4 | 4 | 53 |
| 0 | 1 | 1 | 1 | 23 | 12 | 0 | 0 | 23 | 12 | 19 | 25 | 114 |
| 1 | 1 | 0 | 1 | 0 | 0 | 0 | 0 | 1 | 0 | 0 | 0 | 1 |
| 1 | 1 | 1 | 1 | 4 | 5 | 0 | 0 | 3 | 10 | 7 | 2 | 31 |

Table S12: Combinations of test results from the 8 schools purposively selected for further sample analysis in Leiden when POC-CCA trace was considered positive. 0 in the test results denotes negative and 1 denotes positive by the respective test. The number of children with each test combination is each school is shown on the right hand side. For example, 10 children in school 1 tested negative on all four tests when POC-CCA trace was positive (0-0-0-0 in top row)
